# Caffein an Antidote to Opium

**Published:** 1860-11

**Authors:** 


ART. VIII.—*Caffein as an Antidote in the Poisonous Narcotism of Opium.* By HENRY FRASER CAMPBELL, A.M., M.D. pp. 12. Augusta, 1860.

PROFESSOR CAMPBELL has acted wisely in publishing this paper, inasmuch as it will tend to rouse the attention of the profession of the country to caffein as an antidote for poisoning from opium; an occurrence, unfortunately, too frequent in all parts of the world. After some general remarks upon the nature of this alkaloid, and the effects of coffee, the distinguished author details the particulars of a case of extreme narcotism of opium promptly relieved by artificial respiration and the administration of caffein by injection. The patient, a man, aged twenty-four, had taken at least one ounce and a half of laudanum nearly an hour before, and by the time the stomach-pump could be procured, the respiration had nearly ceased. The quantity of caffein injected into the rectum was twenty grains, and the effects of the remedy became conspicuous in a very short time after its administration. In less than an hour the patient was so much revived as to jerk his arm forcibly from the assistant; the respiration rapidly improved, and, although the drowsiness continued for some time longer, there was no recurrence of coma or of muscular relaxation. Should occasion occur again for its use, Dr. Campbell thinks that he would give caffein in smaller doses, as five grains, repeated until the desired effect was produced.

The following are the author's concluding remarks:—

"The principal object of the present report, however, is only to extend the results of the above remarkable case, wherein the *antinarcotic* effect of the drug had been very apparent; and we therefore desire to dwell no longer on incidental physiological phenomena. If in caffein—so powerful an alkaloid, possessing, in a concentrated form, all the antisoporific virtues of coffee—we have thus found an *antidote* for the narcotic effects of opium, and one which can be applied even in the most extreme states, *by injection*, we must feel that an important extension of its application as a therapeutic agent has been made, and that many lives may be saved hereafter by its use. Reasoning from the result of a single case, it is true, however remarkable that case may be, is, we are aware, always more or less unreliable; but, with the most jealous interpretation of the phenomena, as we observed them, we have been forced to the belief that the means used here acted most powerfully in producing the favorable result. Indeed, we have never witnessed sequences after the administration of a medicinal agent which impressed us more fully with the conviction of *cause and effect*. We would, however, take occasion, in closing, to urge the repetition of the administration of caffein in cases of opium-coma, to a sufficient number of the many which are daily occurring under the eyes of the profession, in order to prove or disprove the validity of our confidence in the remedy."

